# Author Index for Volume 96

**DOI:** 10.1038/sj.bjc.6603845

**Published:** 2007-06-12

Aaltonen, LA 352, 1896

Abbadessa, A 864

Abbott, CM 1613

Abe, D 290

Abnet, CC 172, 1469

Abou El Hassan, MA 450, 937

Ackland, SP 231

Adachi, S 1191

Adami, HO 1475

Adamopoulos, IE 1716

Adatia, R 886

Adena, M 546

Agnantis, NJ 1404

Aguayo, F 1554

Ahlman, H 1656, 1834

Aikou, T 1353

Ainsworth, P 118

Aitini, E 439

Akashi, Y 1532

Akiba, S 1554

Al Murri, AM 891

Alam, SM 1735

Ala-Opas, M 56

Albrektsen, G 1433

Alexeyev, O 137

Alexopoulos, CG 1644

Alfanz, A 485

Allard, A 137

Allasia, C 329

Allègre, G 336, 1314

Almond, J 1489

Al-Mukhtar, A 222

Alston, RD 1760

Altieri, A 1755

Ammerpohl, O 73

Ancona, E 432

Andersen, CL 499, 1896

Anderson, E 575

Anderson, J 856

Anderson, JA 1394

Anderson, SE 1823

Andersson, J 1656

Andersson, T 808

Andrac-Meyer, L 329

Andrews, EJ 262

Angerson, WJ 891

Anuar, D 776

Anwar, MS 104

Aoki, S 631

Aonuma, K 290

Aoyama, N 89

Araidy, S 1101

Argov, S 980

Arkenau, HT 1409

Armstrong, JL 1062, 1675

Arndt, V 828

Arslan, F 1560

Arts, N 1605

Asahina, H 400

Ashley, S 718

Ashworth, A 341

Athanasou, NA 1716

Atherton, J 1855

Atkinson, MJ 801

Aubele, M 801

Audhuy, B 1633

Auer, G 801

Auvinen, A 56

Azzarello, A 769

Babiak, A 1052

Bacher, U 535

Bachmann, M 1293, 1928

Backen, AC 1544

Backus, HHJ 769

Bader, SA 660

Baeuerle, PA 417, 1491

Bafna, UD 1107

Bågeman, E 712

Bailey, H 708

Bain, M 752

Bains, M 1823

Bajetta, E 439

Baker, G 1425

Baldan, A 1778

Baldewijns, MM 1888

Balmer Majno, S 1743

Barbachano, Y 1030

Baril, P 373

Barnabas, RV 514

Barnes, NLP 575

Barone, C 21, 439

Barrett, JC 1436

Bartel, C 157

Bartlett, JMS 801, 1623

Barton, MB 391

Basser, R 1154

Basso, M 21

Bast, A 450, 937

Bastit, L 1166

Bate, SC 44

Bateman, DN 752

Batistatou, A 1404

Baxter, L 226

Beauseigle, D 646

Becker, JC 1540

Bees, N 1030

Begent, RHJ 1862

Beghelli, S 1358

Beirne, DA 44

Belinsky, SA 1278

Belosay, A 1204

Benchimol, S 1425

Benitez, DA 1595

Ben-Izhak, O 1101

Benner, A 82

Benoît, G 336, 1314

Benson, C 29

Beral, V 140, 507

Berchier, MC 1644

Berek, JS 534, 1492, 1817

Berger, W 960

Bergh, J 137

Berghmans, T 1644

Bergmann, JH 1613

Berkhof, J 937, 1234, 1419

Bestor, TH 201

Betts, G 1849

Bezdetnaya, L 944

Bhakta, V 373

Bianchi, S 1253

Bidoli, P 180

Billingham, L 104

Bilous, M 1253

Birch, JM 815, 1265, 1760

Birch-Machin, M 1062

Birkenkamp-Demtroder, K 1896

Birnbaum, D 329

Bishop, DT 357

Bishop, JL 1747

Bishop, PW 95

Blaheta, RA 1699

Blanchard, F 1166

Blaser, MJ 172

Blatt, V 1047

Bleeker, MCG 1419

Bluekens, AM 1888

Bocklage, TJ 1278

Boddy, AV 424, 725, 1675

Bodmer, D 1605

Bogdahn, U 1560

Bonafé, M 1302

Bonanomi, AG 218, 1750

Bonneterre, J 1633

Bonnier, P 329

Borchard, F 1409

Bordonaro, R 864

Borel, M 1684

Borre, M 499

Borthwick, NJ 1879

Boshoff, C 1659

Bosmans, J 1692

Boso, C 432

Bosserhoff, A-K 1560

Boterberg, T 692

Bouchardy, C 1743

Bouchon, B 1684

Boutron-Ruault, M-C 841

Bower, M 732

Boxer, G 1862

Boyd, J 1613

Boyé, K 269

Boyer, M 1154

Bozanovic, T 321

Bradley, G 1425

Brakenhoff, RH 769

Brandes, AA 1047

Braselmann, H 801

Bray, C 1772

Bray, F 1484

Breda, E 1639

Brekelmans, CT 1335

Bremer, E 241

Brenner, H 828, 1329

Bressac-de Paillerets, B 336, 1314

Brewster, AE 708

Brewster, DH 818, 1747

Bridle, H 1904

Brinton, LA 1450

Bröcker, EB 1540

Brockmeyer, AD 1908

Brodie, AMH 1204

Brooks, DR 519

Brown, D 744

Brown, G 1030

Brown, J 591

Brown, R 1623

Brownhill, SC 1914

Brunner, HG 1605

Bruynzeel, AME 450, 937

Bryant, J 226

Bryden, JR 1747

Buchroithner, J 960

Bulk, S 1234, 1419

Bulkmans, NWJ 1419

Bümming, P 1656, 1834

Buncher, CR 970

Bundred, N 1197

Bundred, NJ 575

Burchill, SA 1914

Burger, AM 1223

Burger, CW 1335

Burger, M 1711

Burley, VJ 1139, 1780

Bussink, J 1309

Butt, E 296

Butzow, R 1621

Buzzoni, R 439

Byrne, CD 1747

Cade, JE 1139, 1780

Cai, H 248

Camidge, DR 752

Campbell, M 1197

Candela, M 864

Capelli, P 1118

Cardoso, F 1504

Cargill, A 868

Carpentier-Meunier, S 329

Casali, PG 180

Casas, E 1278

Casieri, P 180

Cassano, A 21

Cassidy, J 29

Castellón, E 1595

Castiglione, G 218, 1750

Castillo, A 1554

Casuccio, A 1828

Caulee, K 373

Cavallo, G 1047

Cebon, J 1154

Ceelen, W 692

Cesteleyn, L 692

C-Gaudreault, R 1684

Chalmers, CR 1112

Chan, A 952

Chan, C 591

Chan, JK 534, 1492, 1817

Chan, KCA 681

Chan, KH 623

Chan, SK 1010

Chan, YH 776

Chander, SK 1368

Chappuis, P 1743

Charafe-Jauffret, E 329

Charalabopoulos, A 1404

Charalabopoulos, K 1404

Charbonnier, F 1166

Charpin-Taranger, C 329

Chatterjee, N 1450

Chatterjee, R 1808

Chaudry, MA 1013

Chautard, E 474

Chelala, C 373

Chen, C-L 591

Chen, C-Y 583

Chen, GG 477

Chen, H-Y 541

Chen, P-S 541

Chen, R 952

Chen, Z 118

Cheng, G 591

Cheng, SH 617

Cheng, WM 623

Cheon, JH 1324

Cherian, J 738

Cheung, MK 534, 1492

Chiappa, A 1118

Chiarion-Sileni, V 432

Chidgey, M 1783

Chieco, P 1302

Chilosi, M 1118

Choi, IK 1514

Choi, YS 1324

Chokkalingam, AP 1475

Chollet, P 1633

Chong, G 1178

Chrétien, Y 336, 1314

Christensen, R 126

Christofori, G 1

Chua, DTT 623

Chuang, S-E 541

Chun, HJ 1514

Chung, SJ 1324

Ciatto, S 218, 1253, 1750

Cinatl Jr, J 1699

Clamp, AR 1544

Clark, DP 952

Clarke, PA 1855

Clavel-Chapelon, F 841

Clavère, P 1633

Cleator, SJ 341

Clegg, A 226

Clementi, M 1595

Clifford, GM 1127

Coebergh, JWW 1767

Colditz, GA 151

Cole, CL 1544

Cole, M 424

Coleman, MP 1135

Coleman, N 667, 1107

Colley, A 391

Collins, L 583

Collon, T 1644

Colozza, M 1504

Communal, Y 1684

Comstock, CES 970

Confortini, M 218

Connolly, NB 567

Contractor, R 445

Conway, DI 818

Cooke, TG 1625

Cookson, JC 1223

Cookson, MM 1520

Cooper, N 1135

Cooper, PD 67

Corazzari, M 1062

Corazzelli, G 864

Cordero, OJ 1568

Corleto, VD 1778

Corner, J 875

Correale, P 1343

Corte, MD 903

Corti, L 432

Costa, DB 399

Coupland, V 1484

Couto, E 1740

Couturier, J 654

Couvelard, A 49

Cox, J 600

Craven, O 38

Crawford, D 1154

Cree, IA 1879

Crijns, APG 1623

Crinò, L 1047

Cronin-Fenton, DP 1462

Crosby, TDL 708

Cruickshank, C 29

Cunningham, D 206, 1178, 1183

Cutts, SM 1667

Cuzick, J 110

Dai, F 1223

Dalès, J-P 329

Damelin, M 201

Damianovich, D 546

Dandrea, M 1358

Danforth, KN 151

D'Argento, E 21

Davidson, R 718

Davies, BG 1265

Davies, MC 1808

Dawsey, SM 172

Dawson, C 1783

de Azambuja, E 1504

de Bock, GH 1335, 1623

De Bono, J 29

De Bruïne, AP 1888

de Castro Jr, G 1504

de Cremoux, P 269

de Graeff, P 1623

de Hullu, JA 1335

de Jong, S 1623

De Mulder, PH 1309

de Pinieux, G 269

De Rosa, V 1639

de Schipper, FA 1234

Dearling, JLJ 1862

Deavers, M 1083

Degoul, F 1684

Del Bianco, P 432

Del Vecchio, MT 1343

Delaloye, J-F 1743

Demetter, P 692

d’Enghien, CD 654

Denoux, Y 336, 1314

Denzinger, S 1711

Desreux, J 841

Devens, F 82

Dewar, R 162

Dexter, T 341

Dhillon, H 1154

Di Bartolomeo, M 439

Di Bisceglie, M 1343

Di Carlo, V 1358

Di Fiore, F 1166

Di Girolamo, N 783

Di Leonardo, G 21

Di Maio, M 1639

Diamandis, EP 362

Dickman, B 1154

Dickman, PW 519

Diéras, V 654

Dillner, J 514

Dimri, GP 126

Direkze, S 182

Dittrich, C 559

Dixon, AK 882

Dixon, JM 1802

Doerfelt, A 1560

Doerr, HW 1699

Doherty, V 1772

Doherty, VR 832

Doorn, L 1348

Doughty, JC 891

Dowling, A 1154

Downing, A 836

Dowsett, M 341

Dresselaers, T 758

Drummond, M 143

du Bois, A 306

Dubeau, L 1083, 1908

Dubois, L 758

Dubrova, YE 1265

Dudakia, D 357

Dudderidge, TJ 1384

Duggan, C 110

Duivenvoorden, WCM 1526

Dumas, D 944

Duran, I 178

Durbecq, V 1504

Dutrillaux, B 269

Dyck, J 1692

Easton, D 357

Ebert, MP 95

Ebisawa, S 1707

Ebner, R 1293, 1928

Eccles, D 718

Eckardt, A 408

Eckel, F 896

Eden, TOB 1265, 1760

Edwards, RE 248

Eeles, R 718

Efremidis, A 1644

Eggert, AO 1540

Eisen, T 1904

Eisen, TG 44

El Assoued, Y 49

el Galta, R 1904

El-Emir, E 1862

Eley, HL 1216

Elgh, F 137

Ellershaw, C 725

Ellis, IO 1092

El-Shikh, S 1092

El-Zaatari, M 1855

Enzinger, T 1409

Errington, J 424, 725

Eskens, FALM 1159, 1348

Esmy, PO 738

Espinosa, AV 16, 1313

Etoh, T 1723

Evans, A 1772

Evans, DGR 718

Ewertz, M 1462

Eymard, J-C 1633

Fabre, A 841

Faedi, M 864

Fairley, L 1484

Falk, S 551

Fallon, M 868

Fan, J-H 172

Fanshawe, TR 1384

Fargeot, P 1633

Farinati, F 1778

Farnie, G 575

Fayette, JM 738

Featherstone, C 391

Febbraro, A 1639

Feder-Mengus, C 1072

Fedida, S 980

Feltbower, RG 1147

Fenwick, K 341

Ferko, N 143

Ferlicot, S 336, 1314

Fernandez-Salguero, PM 1595

Ferrandina, G 1639

Ferrario, E 439

Ferreira, CG 769

Ferrera, P 1828

Ferreri, AJM 864

Fimia, GM 1062

Findlay, M 1154

Finlayson, AR 1747

Fioretta, G 1743

Fischer, J 960

Fishman, D 980

Flaherty, KT 445

Flanagan, JM 183

Flemer Jr, S 1520

Fodstad, O 609

Foot, ABM 424, 725

Forastiere, A 952

Ford, CE 808

Forget, M-A 646

Forman, D 836, 1484

Forman, MR 1436

Formica, V 1178

Formicola, R 1358

Fornander, T 801

Forni, M 1743

Forsberg, G 567

Fosså, A 1442

Fosså, SD 1442

Foster, C 718, 875

Foster, NA 667

Foster, PA 762, 1368

Fournier, A 841

Foweraker, K 1001

Frame, S 752

Franceschetti, I 1118

Franceschi, E 864, 1047

Franceschi, S 1127

Francini, G 1343

Franco, EL 143

François, A 944

Franklin, WA 1278

Fransen Daalmeijer, NC 1234

Frappart, L 738

Frebourg, T 1166

Freedman, ND 1469

French, KM 514

Fréneaux, P 654

Friedman, E 11

Friger, M 980

Fritschi, L 701

Fu, J 477

Fujimoto, J 1735

Fukagawa, T 1723

Fukuoka, M 1532

Fukushima, M 231

Fumoleau, P 1633

Funada, Y 1191

Furuse, J 1650

Fushiki, S 255

Fussel, M 1293, 1928

Gaarenstroom, KN 1335

Gabra, H 1613

Gabriel, FG 1879

Gaco, V 1409

Gad, S 336, 1314

Galais, MP 1166

Gallagher, JT 1544

Gallimore, A 1849

Gallivan, S 143

Gallo, C 1639

Ganesan, R 104

Gangeswaran, R 373

Garas, G 1154

Garcia, S 329

Garcia-Closas, M 1450

Garcia-Manero, G 1153

García-Muñiz, JL 903

Garne, JP 1462

Garnett, GP 514

Gartenhaus, RB 1223

Gayther, SA 321

Gebhardt, S 241

Gebski, V 1154

Gehrmann, M 241

Georgitsi, M 352

Gescher, AJ 248

Ghaneh, P 1183

Ghosh, S 1072

Gianella-Borradori, A 29

Gibbens, IM 44

Gibbons, CLMH 1716

Giele, H 1716

Gilbertson, RJ 6

Gill, PS 1083

Gillet, J-P 401

Gilthorpe, MS 836

Giner, V 1644

Giommoni, E 1043

Giotopoulos, G 1001

Giovannetti, E 769

Giovannucci, E 169

Giraud, S 336, 1314

Gires, O 417, 1491

Glauche, I 677

Glynne-Jones, R 551

Gneist, M 559

Godde, F 1052

Godfrey, A 1659

Godin-Ethier, J 646

Godkin, A 1849

Gök, M 1234

Goldbohm, RA 510

Gollnick, SO 1839

Gómez, C 1568

Gomez, D 222

Gompel, A 841

González, LO 903

Goossens, M 1605

Gore, ME 44

Gorman, M 1729

Gotti, G 1343

Goudier, M-J 1633

Grabowska, AM 464, 1394, 1855

Graham, A 1613

Grazzini, G 218, 1750

Greaves, P 248

Green, A 1092

Green, J 140

Green, M 1154, 1484

Greenwood, DC 1139, 1780

Greggi, S 1639

Gregor, M 1409

Gridley, G 1475

Griep, U 73

Griffin, M 1001

Griffioen, AW 1888

Griffiths, EA 95, 1377

Grifoni, R 1043

Grimes, MJ 1278

Grobholz, R 912

Grönberg, H 137

Gröne, EF 82

Gröne, H-J 82

Groome, NP 1808

Gross, L 104

Grosso, D 1047

Gruenagel, H-H 1409

Grunewald, TGP 296

Grutzmann, R 73

Guarnieri, A 1343

Guastalla, J-P 1633

Guerra, G 1729

Guillemin, F 944

Guillou, PJ 1112

Gullane, P 1425

Gullick, WJ 284, 1010

Gunaratnam, Y 875

Guo, J 583

Guo, YQ 623

Gustavsson, C 137

Guyader, C 269

Ha, TC 1796

Haass, NK 445

Hackshaw, A 1926

Haegert, D 793

Haferlach, C 535

Haferlach, T 535

Hagemann, T 321

Hakama, M 56

Hale, JP 667

Hall, BM 591

Hall, J 1623

Hall, S 1057

Hamada, C 1170

Hamady, ZZR 222

Hammel, P 49

Han, HC 776

Han, T 126

Hanby, A 110

Hankinson, SE 151

Harada, H 1871

Harada, T 373

Harding, NJ 815

Hargreaves, C 1154

Harrison, DJ 660

Hartmann, A 1711

Hartmann, JT 1409

Hasenberg, C 1699

Hasenclever, D 241

Hass, HG 1409

Hasuo, T 89

Hau, P 1560

Haug, U 828, 1329

Hawkins, MM 1439

Hawkins, NJ 783

Hawkins, RE 567

Hayashi, S 793

Heaney, RP 1008

Hebeda, KM 1605

Heberer, M 1072

Hecht, JL 151

Hedlund, G 567

Hefler, L 485

Hemminki, K 1272, 1740, 1755

Hengstler, JG 241

Hentic, O 49

Hepworth, SJ 815

Herlyn, M 445

Hermes, M 241

Hermsen, BBJ 1335

Hervé, JM 336, 1314

Hettiaratchi, A 783

Hetzel, J 1052

Hetzel, M 1052

Heuch, I 1433

Hibberd, C 868

Hidalgo, M 952

Higgins, B 1879

Hilakivi-Clarke, L 134

Hill, C 1155

Hill, DS 1062

Hills, M 341

Hind, D 1025

Hinnen, P 1159

Hinnis, AR 639

Hiraoka, M 1871

Hirohashi, S 1404

Hirokawa, M 1549

Hisada, S 1532

Hiyama, E 1020

Hiyama, K 1020

Ho, L 110

Ho, M 776

Hochhaus, A 912

Hoebe, EK 231

Hoffmeister, M 828

Hofheinz, R-D 912

Hollema, H 1623

Hollenbeck, AR 1469

Hollywood, D 1587

Holte, H 1442

Homma, S 290

Hong, C-C 541

Honig, A 296

Hoogerbrugge, N 1605

Hooper, S 321

Hopkinson, J 875

Hopwood, P 718

Horak, P 485

Horan, G 882

Horie, N 255

Horisberger, K 912

Horn, M 677

Horvat, R 485

Horwich, A 529

Hosono, S 314

Houben, R 1540

Houlston, RS 1904

Houssami, N 1253

Houtmeyers, P 692

Howarth, KM 1729

Howell, A 1197

Hsieh, C-J 1409

Hsieh, F-C 591

Hsing, AW 1475

Hu, JM 1817

Hu, XF 918

Huddart, R 357

Hudson, E 708

Hughes, J 464

Hughes, SA 44

Huh, WK 1480

Huhalov, A 1862

Huitema, ADR 559

Hummer, A 1823

Hung, M-C 541

Hunt, JE 783

Hurren, J 1879

Husain, A 534, 1492, 1817

Hussain, A 1265

Hussain, SA 104, 1310

Iacopetta, B 701

Ikeda, H 1650

Ikeda, J-I 986

Ikeda, M 986, 1650

Ilariucci, F 864

Ilson, DH 1823

Imawari, M 492

Ingvar, C 712

Innocente, R 432

Ino, K 314

Intravaia, G 1828

Intrivici, C 1343

Iop, A 439

Iravani, M 341

Irish, J 1425

Irthum, B 474

Isa, L 439

Ishii, H 1650

Ishikawa, K 1723

Ito, H 492

Ito, K 993

Ito, Y 1549, 1650

Iwasa, T 1532

Izawa, T 457

Jack, R 886

Jack, WJL 1802

Jackson, A 189

Jacobelli, S 439

Jacobsen, J 1462

Jacquemier, J 329

Jager, MJ 1879

Jahan, I 1735

James, ND 104

Janssen, O 73

Jass, J 793

Jayne, DG 1112

Jayson, GC 189, 1544

Jazag, A 993

Jeen, YT 1514

Jenkins, D 143

Jenkins, JT 213

Jennings, N 1083

Jernström, H 712

Ji, J 1272

Ji, MF 623

Jiang, W 575

Jimeno, A 952

Johansson, JE 1475

John, TG 1037

Johnson, DH 1278

Johnson, P 1879

Jonas, D 1699

Jones, C 341

Jones, ME 850

Joos, S 82

Judde, J-G 269

Judson, I 29

Jung, HC 1324

Junquera, S 903

Juusela, H 56

Kabat, GC 845

Kabingu, E 1839

Kagami, Y 1650

Kähler, G 912

Kajiyama, H 314

Kalff, V 1154

Kalina, M 1526

Kallioniemi, A 1258

Kalthoff, H 73

Kamangar, F 172

Kamba, T 1788

Kammerer, U 296

Kamoshida, S 277

Kaneko, K 492

Kang, D 1475

Kang, MK 126

Kapp, DS 534, 1492, 1817

Karamouzis, MV 1924

Karhu, A 352

Karhu, R 1258

Katagiri, A 492

Katai, H 1723

Katalinic, A 157

Katki, H 172

Kato, M 631

Kato, T 1170

Katsaros, D 362

Kauczok, CS 1540

Kawashima, M 1650

Kaye, P 1855

Kaye, S 29

Kelly, G 373

Kelly, J 583

Kelly, JD 1384

Kelsen, D 1823

Kémény, J-L 474

Kerbrat, P 1633

Kern, W 535

Kerr, GR 1802

Kessler-Icekson, G 1667

Kets, CM 1605

Khalil, T 474

Khan, N 1554

Khandelwal, A 1204

Khoo, SK 336, 1314

Kikkawa, F 314

Kilany, S 567

Killeen, SD 262

Killian, A 1166

Kim, CW 1324

Kim, JS 1324, 1514

Kim, RH 126

Kim, SG 1324

Kim, S-H 583

Kim, SJ 126

Kim, YH 1514

Kimura, S 255

Kincaid, E 952

Kindblom, L-G 1656

King, AAJ 559

Kinoshita, I 400

Kinsey, SE 1147

Kirk, J 391

Kiserud, CE 1442

Kitamura, H 1707

Kleiber, G 1083

Klint, A 519

Kloppel, G 73

Klump, B 1409

Knudsen, KE 970

Kobayashi, S 399

Kodaira, S 1170

Koelbl, H 241

Koh, W-P 821

Kohgo, Y 457

Kohli, M 143

Kohlmann, A 535

Koizumi, K 457

Kommoss, F 306

Kommoss, S 306

Komori, Y 277

Konishi, F 383

Konishi, K 492, 986

Konstantinopoulos, PA 1924

Kontula, O 514

Korbelik, M 67

Koriyama, C 1554

Kosaka, Y 1723

Koscielny, S 1155

Kotani, Y 1191

Kotsopoulos, J 118

Koumi, A 1659

Ko"nig, H-H 1052

Krag, DN 1520

Krainer, M 485

Krasnoperov, V 1083

Kraus-Tiefenbacher, U 912

Krug, T 1237

Kruhøffer, M 1896

Krupa, K 1625, 1802

Kruyt, FAE 450

Kubo, T 255

Kubota, Y 492

Kuipers, EJ 1767

Kulesza, P 952

Kumar, SR 1083

Kumari, R 1855

Kumekawa, Y 492

Kunitoh, H 1498

Kunkler, IH 1802

Kuo, I-H 541

Kuo, M-L 541

Kurahashi, T 492

Kusano, M 631

Kushima, M 492

Kuukasjärvi, T 1258

Kvåle, G 1433

Ladds, S 744

Lae, M 654

Lafitte, JJ 1644

Lai, PBS 477

Laiho, P 1896

Lambe, M 519

Lambin, P 758

Lamelas, ML 903

Lamy, A 1166

Lancashire, ER 1439

Landriscina, M 21

Landuyt, W 758

Lannigan, A 891

Lapointe, R 646

Larkin, JMG 44

Larsimont, D 1504

Larsson, SC 1457

Lash, TL 1462

Laskey, RA 1107

Lassus, H 1621

Laster, Z 1101

Latte, G 864

Lau, K-M 617

Lau, KW 1284

Lau, SC 1544

Lau, SK 808

Lau, YS 1716

Launonen, V 352

Lavaut, M-N 329

Lavelle, K 1197

Lawler, M 1587

Le Pessot, F 1166

Lear, J 523

Leclercq, N 1644

Lecomte, J 1644

Ledermann, JA 1311

Lee, CK 776

Lee, I-M 1009

Lee, JT 445

Lee, RJ 1747

Lee, SAKW 617

Lee, SP 617

Leese, MP 1368

Lefèvre, SH 336, 1314

Legrier, M-E 269

Lehr, H-A 241

Lehtinen, M 514

Leiserowitz, GS 534, 1492

Leite, V 1237

Leitner, A 912

Leitzmann, MF 1469

Lemoine, NR 373

Lemos-González, Y 1568

Lester, JF 708

Leung, HY 1384

Levitt, G 226

Lévy, P 49

Levy-Lahad, E 11

Li, D 89

Li, H 1796

Li, J 918

Liang, JS 623

Liau, S-S 993

Lichter, P 82

Lickliter, JD 600

Lieblein, JC 591

Ligtenberg, MJL 1605

Lin, H 600

Lin, J 591

Lindhofer, H 1013

Linehan, WM 403

Linger, R 357

Lissowska, J 1450

Liu, J 1871

Liu, S 523

Ljubic, A 321

Lo, KW 617

Lo, YMD 681

Lo Vullo, S 439

Loddo, M 1384

Lodge, JPA 222

Löf, M 134

Loftus, B 1587

Lonardi, S 1047

Londesborough, P 110

Looman, CWN 1767

Lortholary, A 1633

Lorusso, D 1639

Lovat, PE 1062

Lu, X 196

Luckett, JCA 639

Lugli, A 793

Lunec, J 762

Luscombe, CJ 523

Lutchman Singh, K 1808

Lynch, TH 1587

Lyon, MD 1480

Määttänen, L 56

Macdonald, G 1154

MacKay, A 341

Mackay, J 718

MacKie, RM 1772

MacMillan, C 1425

Macpherson, LMD 818

Madelmont, JC 1684

Maekawa, T 255

Maemura, K 1353

Magrini, E 1047

Mahlke, M 241

Mahon, PC 373

Maisonneuve, P 1118

Makino, R 492, 631

Malcles, M-H 1659

Malik, HZ 222

Mangione, S 1828

Man-i, M 986

Manno, P 864

Mano, MS 1504

Mansilla, F 1896

Mantellini, P 218, 1750

Marchal, S 944

Mariani, L 439

Mark, SD 172

Marklund, I 137

Marosi, C 960

Marrache, F 49

Marsili, S 1343

Marteau, TM 1057

Martikainen, P 56

Martin, A 143

Martin, I 1072

Martin, J 646

Martin, SG 1092

Martinelli, F 218

Martinez, NR 600

Mason, W 864

Masood, R 1083

Massuger, LF 1335

Masuda, Y 631

Masullo, M 1118

Mataki, Y 1353

Matakidou, A 1904

Matsui, T 255

Matsumoto, K 290

Matsuura, N 986

Mattes, MJ 928

Mauer, S 609

Maughan, TS 551, 708

Mazza, E 864

Mazzarotto, R 432

McArdle, CS 891

McCarron, J 600

McClelland, CM 284

McCormick, D 1879

McCracken, SR 1384

McDonald, DM 1788

McDonald, JW 875

McDowell, H 725

McGarrigle, HH 1808

McGrath, H 29

McGrath, SM 1377

McKenzie, G 783

McKinney, PA 815, 818, 1147

McMahon, AD 818

McMillan, DC 891

McVey, R 1544

Meadows, HM 551

Medina, CA 445

Meert, AP 1644

Mehta, J 1204

Meijer, CJLM 1234, 1419

Meis-Kindblom, JM 1656

Méjean, A 336, 1314

Mellemgaard, A 886

Mellon, JK 762

Menestrina, F 1118

Mengesha, A 758

Mercadante, S 1828

Mercalli, A 1358

Mercatelli, A 1043

Merino, AM 903

Merkle, K 1692

Mertens, D 82

Mesrine, S 841

Miao, J 477

Michaelis, M 1699

Michaud, DS 169

Michel, P 1166

Michelagnoli, MP 1147

Michiels, S 1155

Michor, F 679

Michot, F 1166

Micksche, M 960

Mikeljevic, JS 836

Miki, Y 383

Miller, AB 845

Mimori, K 1723

Minoguchi, M 457

Minsky, BD 1823

Miot-Noirault, E 1684

Miselli, F 180

Mitry, E 49

Mitsudomi, T 857, 1191

Miyaji, Y 383

Miyauchi, A 1549

Mizukami, Y 457

Mohammed, RAA 1092

Mok, YJ 1514

Mokhtari, K 474

Molinié, V 336, 1314

Møller, B 1484

Møller, H 529, 886, 1484

Monden, M 986

Monnier, A 1633

Montanaro, L 1302

Montcuquet, P 1633

Montgomery, DA 1625, 1802

Mooij, TM 1335

Moore, MJ 178

Moran, A 1197, 1760

Morgan, MA 408

Mori, M 1723

Morizane, C 1650

Morohara, K 631

Moroose, R 1587

Morran, CG 213

Morris, M 701

Morrison, PJ 718

Mounetou, E 1684

Mourits, MJ 1335

Mowbray, M 832

Muehlenweg, B 262

Mukherjee, G 1107

Mukherjee, S 708

Munro, A 801

Muralidhar, B 1107

Muramoto, T 492

Murata, H 255

Murray, PG 104

Mustea, A 241

Muttukrishna, S 1808

Mwanahamuntu, MH 1480

Nagasawa, T 290

Nagler, RM 1101

Nakagawa, K 1532

Nakamura, K 457

Nakanishi, A 383

Nakanishi, K 290

Nakanishi, Y 1404

Nakano, Y 457

Nakazato, H 1170

Namer, M 1633

Narita, M 686

Narod, SA 118

Nathanson, KL 445

Natsheh, I 1699

Navarro Silvera, SA 845

Nawa, A 314

Neal, DE 1384

Neckers, L 403

Negoro, S 1191

Negri, T 180

Nehls, O 1409

Neoptolemos, JP 1183

Neri, B 1043

Neri, D 1862

Newbery, HJ 1613

Newman, SP 1368

Neyroud-Gaspar, I 1743

Ng, MH 623

Ng, MHL 617

Ng, SP 623

Ng, SS 776

Nga, ME 776

Ngan, CY 986

Nicholson, JC 667

Nickl-Jockschat, T 1560

Nicol, AJ 600

Nicolson, M 1772

Nieda, M 600

Niessen, HWM 937

Nilsen, TIL 1436

Nilsson, B 1656, 1834

Nilsson, O 1656

Ninane, V 1644

Nishikawa, T 457

Nishino, N 631

Njar, VCO 1204

Noda, H 383

Noma, H 1353

Nordle, Ö 567

Nørgaard, M 1462

Norman, AR 1030, 1178

Nos, C 654

Novelli, MR 1729

Nowak, A 1154

Nowell, GM 725

Nozawa, H 492

Nudelman, A 1667

Nutt, JE 762

Nyren, O 1475

Oates, J 1178

Oba, K 1170

Obermann, EC 1711

O'Connor, JPB 189

Odén, A 1834

O'Donnell, A 29

O'Donnell, PH 177

Oertli, D 1072

Ogura, M 1871

Oh, SC 1514

Ohashi, Y 1170

Okabe, A 1707

Okada, M 1707

Okamoto, I 1532

Okayasu, R 1707

Okech, T 1409

Okello, C 1484

Oki, A 290

Okumura, T 457

Okusaka, T 1650

Olivier, RI 1335

Omori, Y 89

O’Neill, G 213

Ono, K 1532

Oren, L 980

Ørntoft, TF 499, 1896

Orsenigo, M 180

Osann, K 534, 1492, 1817

Osman, A 1001

O’Toole, D 49

Oudard, S 269

Overbeek, LIH 1605

Owczarczak, B 1839

Pace, A 864

Paesmans, K 758

Paesmans, M 1504, 1644

Páez de la Cadena, M 1568

Paillot, B 1166

Paish, EC 1092

Palmer, DH 104, 1310

Palmer, K 38

Palmer, RD 667

Pandolfi, S 1302

Pantaleo, P 1043

Pantalone, D 1043

Paoletti, C 1043

Papavassiliou, AG 1924

Pappin, C 732

Parham, GP 1480

Parikh, B 1808

Park, N-H 126

Park, SS 1514

Parker, GJM 189

Pärssinen, J 1258

Pascucci, A 1343

Pastorek, J 104

Patel, A 1808

Patel, JB 1204

Patel, N 886

Patel, PM 44, 567

Patnick, J 140

Pattyn, P 692

Paul, J 1623

Pearson, ADJ 424, 725

Pearson, DG 725

Peat, I 1001

Pedley, RB 1862

Peeters, M 692

Pelletier, S 646

Pelosi, G 1118

Peplonska, B 1450

Perez-Perez, GI 172

Pero, SC 1520

Perrone, F 1639

Perry, AS 1587

Perry, J 864

Perunovic, B 104

Peschos, D 1404

Pession, A 1047

Peters, GJ 61, 231, 769

Pfisterer, J 306

Phatak, P 1223

Phillips, DR 1667

Phillips, RKS 1729

Piacentini, M 1062

Piacenza, C 180

Piccart-Gebhart, MJ 1504

Pichler, J 960

Picot, J 226

Piemonti, L 1358

Pierga, J-Y 341, 654

Pierotti, MA 180

Pietsch, M 1052

Pignata, S 1639

Pigozzo, J 432

Pilarsky, C 73

Pilch, H 241

Pilotti, S 180

Pils, D 485

Pinedo, HM 769

Pinotti, G 439

Pinter, A 485

Pirker, C 960

Pisano, C 1639

Pizer, B 725

Platell, C 701

Platz, EA 169

Plumb, M 1001

Polak, ME 1879

Poll, A 118

Poole, CJ 104

Poon, A 1154

Poppleton, H 6

Porchia, L 16, 1313

Porschen, R 1409

Portlock, J 744

Post, S 912

Poston, G 1112

Potter, BVL 1368

Poupon, M-F 269, 769

Powe, DG 1855

Powell, J 708

Powles, T 341, 732

Pozo-Guisado, E 1595

Pozzo, C 21

Prakash, K 836

Prasad, KR 222

Prasad, R 1112

Price, PM 95, 1377

Prins, H-J 61

Pritchard, KI 1011, 1781

Pritchard, SA 95, 1377

Pritchard-Jones, K 1493, 1927

Prowatke, I 82

Pugh, CW 1284

Pukkala, E 514

Puppa, G 1118

Purohit, A 1368

Qiao, Y-L 172

Queuniet, AM 1166

Quirino, M 21

Rachet, B 1135

Rafique, A 882

Rajaganeshan, R 1112

Rajkumar, R 738

Ralston, S 1489

Ramachandran, S 523

Ramsey, KD 1839

Rapiti, E 1743

Rapley, EA 357

Raspe, H 157

Rasschaert, M 1692

Ratain, MJ 177

Ratcliffe, PJ 1284

Raval, RR 1284

Ravaud, P 49

Raynaud, F 29

Rayson, D 162

Raza, SA 1127

Rea, DW 104, 1310

Read, H 744

Recaldin, E 439

Redfern, CPF 1062, 1675

Redmond, HP 262

Reed, MJ 1368

Reed, MW 1025

Rees, M 1037

Reeves, M 1265

Reibenwein, J 485

Reid, E 1057

Reinthaller, A 485

Reis-Filho, JS 341

Relja, B 1699

Reni, M 864, 1358

Rephaeli, A 1667

Reschner, A 1072

Reulen, RC 1439

Reuter, CWM 408

Revelo, MP 970

Reynolds, G 104

Rhodes, B 744

Richard, S 336, 1314

Richards, M 1147

Rickinson, AB 617

Ridder, R 306

Rieber, EP 1293, 1928

Ring, J 1154

Ring, SM 1265

Ringel, MD 16, 1313

Riva, N 1639

Roberts, I 667

Roberts, S 1154

Robinson, D 529

Robson, J 882

Robson, MP 1862

Roché, H 1633

Roche, M 140

Roddam, AW 507

Rodrigues, RF 1237

Rodríguez, JC 903

Rodríguez-Berrocal, FJ 1568

Roeder, I 677

Rohan, TE 845

Romundstad, PR 1436

Rookus, MA 1335

Roque, L 1237

Rose, C 712

Roskams, T 1888

Rosner, BA 151

Rotella, V 1043

Rothenbacher, D 1329

Rougier, P 49

Rowan, S 1760

Roy, C 49

Roy, D 1908

Rozendaal, L 1234, 1419

Rubeca, T 218, 1750

Rudd, MF 1904

Ruf, P 1013

Rukin, NJ 523

Ruol, A 432

Rush, R 868

Russell, ST 1216

Ruszniewski, P 49

Ruutu, M 56

Sabnis, G 1204

Sabokbar, A 1716

Sabourin, JC 1166

Saeger, H-D 73

Sahasrabuddhe, VV 1480

Saint-Jacques, N 162

Saji, S 1170

Sakabe, T 255

Sakaguchi, H 1735

Sakamoto, J 1170

Sakoda, LC 1450

Sakuma, T 1191

Sakurai, Y 277

Sales, K 1013

Salter, J 341

Salto-Tellez, M 776

Sandin, S 134

Sankaranarayanan, R 738

Sano, T 1723

Sansone, P 1302

Sappino, A-P 1743

Sarbia, M 1409

Sarkar, R 1112

Sasako, M 1723

Sasisekharan, R 1315

Sastre-Garau, X 654

Sato, E 1735

Satoh, T 290, 1532

Satouchi, M 1191

Saunders, MP 38

Savage, PM 732

Savelli, V 1343

Saxby, M 523

Scambia, G 1639

Scarpa, A 1358

Scehnet, J 1083

Schackert, G 1293, 1928

Schackert, HK 1293, 1928

Schaefer, P 1743

Schalkwijk, C 937

Schatzkin, A 1469

Scherpereel, A 1644

Schiffer, IB 241

Schiller, JH 1278

Schindler, D 296

Schinzari, G 21

Schmalix, W 262

Schmid, BC 485

Schmid, P 732

Schmid, RM 896

Schmidt, CW 600

Schmidt, D 306

Schmidt, M 241

Schmitz, M 1293, 1928

Schneider, DT 667

Schniewind, B 73

Schnittger, S 535

Schreer, I 157

Schrijvers, D 1692

Schumacher, U 609

Schwind, S 1293, 1928

Sciandivasci, A 1343

Scopece, L 1047

Scorilas, A 362

Scott, AM 1154

Scott, D 1613

Scott, N 1112

Scotting, PJ 1855

Sculier, JP 1644

Sebag-Montefiore, D 551

Seckl, MJ 732

Segal, S 980

Segditsas, S 1729

Seidlitz, E 1526

Seiler, CM 1329

Sekimoto, M 986

Sekine, E 1707

Sellar, GC 1613

Semba, S 89

Sengupta, S 1315

Senner, V 1293, 1928

Seo, JH 1514

Sergio, A 1778

Sesboüé, R 1166

Seshimo, I 986

Sesso, HD 1009

Sham, JST 623

Shan, SJC 362

Shanthakumary, S 738

Sharpe, M 868

Shaw, DM 567

Sheikh, HY 38

Shenton, G 1147

Shepard, CR 1246

Shepherd, JH 321

Sherlaw-Johnson, C 143

Sherman, ME 1450

Shibata, K 314

Shibata, T 1871

Shimada, T 1191

Shimomura, R 277

Shin, JY 534, 1492, 1817

Shin, K-H 126

Shin, SW 1514

Shinchi, H 1353

Shirasaka, D 89

Short, D 732

Shukla, GS 1520

Shuyama, K 1554

Sickmann, A 296

Sieber, OM 1729

Sierro, S 1849

Siersema, PD 1767

Sigal-Zafrani, B 654

Silverman, D 1475

Silye, R 960

Simon, H 1633

Simonsson, T 1834

Simpson, P 341

Singh, G 1526

Singh, J 1083

Singh, N 321

Sion-Vardy, N 980

Sipos, B 73

Sirohi, B 1178

Siu, LL 178

Sjölund, K 1656, 1834

Sjursen, A 38

Slettenaar, VIF 321

Smalley, KSM 445

Smeets, P 692

Smith, IE 341

Smith, NJ 1030

Smith, V 1223

Smits, KM 510

Snijders, PJF 1234, 1419

Song, IS 1324

Sonzogni, A 1118

Sood, AK 1083

Soong, R 776

Soprano, DR 1204

Sordi, V 1358

Sørensen, FB 1896

Sørensen, HT 1462

Sørensen, KD 499

Sotiriou, C 1504

Spagnoli, GC 1072

Span, PN 1309

Spannuth, WA 1083

Spiegl-Kreinecker, S 960

Spina, M 864

Spooner, D 104

Stacchiotti, S 180

Ståhle, E 519

Stanley, M 1320

Stark, J 818

Starling, N 206, 1183

Stebbing, J 732

Stegmaier, C 1329

Stein, RC 1808

Steiner, E 241

Stelitano, C 864

Stenman, U-H 56

Stern, PL 567

Stevanovic, S 1293, 1928

Stevens, A 104

Stevens, MFG 1223

Stevens, RJ 507

Steward, WP 248, 1312

Stidley, CA 1278

Stiller, C 1493

Stockler, M 1154

Stockton, DL 752, 832

Stoeber, K 1384

Stoehr, R 1711

Stolz, DB 1246

Storci, G 1302

Storey, D 868

Strange, RC 523

Stratton, MR 357

Strickland, A 1154

Stringer, JSA 1480

Strong, V 868

Su, J-L 541

Sudarshan, S 403

Sullivan, A 196

Sullivan, I 226

Sultana, A 1183

Sun, C-L 821

Sun, Q 1554

Sun, X-D 172

Sundquist, J 1272

Sung, HJ 1514

Sur, HY 1514

Suzlovich, Z 980

Suzuki, K 1498

Suzuki, M 277, 1532, 1707

Sweep, FCGJ 1309

Swerdlow, AJ 850

Swift, RI 1030

Swindell, R 38

Symonds, RP 1001

Szeszenia-Dabrowska, N 1450

Taddei, A 1043

Tait, D 1183

Tajima, Y 631

Takada, Y 1191

Takano, T 1549

Takao, S 1353

Takemasa, I 986

Takeshita, H 255

Talbot, IC 1729

Tallini, G 1047

Tamaya, T 1735

Tamborini, E 180

Tammela, TLJ 56

Tamura, K 1532

Tanguay, S 646

Tanner, B 241

Tanno, S 457

Tarasenko, I 1667

Tarasenko, N 1667

Taylor, C 1914

Taylor, EF 1139, 1780

Taylor, GA 1675

Taylor, GM 1265

Taylor, PR 172

Tchirkov, A 474

Tebbutt, NC 546

Teh, BT 336, 1314

Tekkis, PP 1037

Temme, A 1293, 1928

Temmink, OH 61, 231

ten Hoor, KA 1623

Teng, NN 534, 1492, 1817

Terauchi, M 314

Terracciano, L 793, 1072

Terry, G 110

Thara, S 738

Theys, J 758

Thiéry, J-P 654

Thies, A 609

Thijssen, VL 1888

Thiounn, N 336, 1314

Thirlwell, C 1729

Thomas, HD 1675

Thomas, HJW 1729

Thomas, J 1802

Thomas, K 44

Thompson, P 1154

Thomson, D 1772

Thoresen, S 1433

Thornton, CM 667

Tian, K 1579

Tian, Q-H 477

Tian, Y-M 1284

Tilby, MJ 725

Tilden, D 206

Tilney, HS 1037

Tisdale, MJ 1216

Tobias, A 1855

Todd, C 1197

Tomassi, O 432

Tomlinson, IPM 1729

Tomlinson, VAL 1613

Tonelli, P 1043

Toogood, GJ 222

Tornillo, L 793

Tørring, N 499

Torun, E 450

Tosoni, A 1047

Toyoki, H 1735

Trachsel, E 1862

Trauzold, A 73

Tremblay, S 1425

Trigila, N 21

Trunk, MJ 306

Tsai, Y-H 1659

Tsanou, H 1404

Tsao, MS 808

Tsong, WH 821

Tsujino, T 986

Tsunoda, H 290

Tsutsumi, Y 277

Tucker, K 391

Tudur Smith, C 1183

Tuech, JJ 1166

Turcotte, S 646

Twelves, C 29

Twohig, J 1849

Tworoger, SS 151

Uchihori, Y 1707

Ueno, H 1650

Ukoumunne, OC 1057

Uno, K 290

Uramoto, H 857

Urata, Y 1191

Urban, R 534, 1492, 1817

Urbini, B 1047

Uyama, I 277

Vago, P 474

Vahteristo, P 352

Valentine, HR 95, 1377

Valerio, M 1118

Valle, JW 38

Vallis, KA 118

van Beurden, M 1335

van Blankenstein, M 1767

van Boven, HH 1335

Van Cutsem, O 1644

Van Damme, N 692

Van den Brande, J 1692

van den Brandt, PA 510

Van den Broek, M 1849

Van den Eynden, GG 1888

van der Born, K 231

van der Gaast, A 1348

van der Looij, E 1605

van der Vijgh, WJF 450, 937

van der Zee, AGJ 1623

van Dongen, GAMS 1862

van Gameren, EC 1348

van Gelderop, E 61

van Hasselt, CA 617

Van Hecke, P 758

van Houten, VMM 769

van Kemenade, FJ 1234, 1419

van Krieken, JHJM 1605

Van Laere, SJ 1888

van Meerten, E 1348

van Poppel, H 1888

Van Trappen, POA 321

Vandervalk, S 918

Vasiliu, V 336, 1314

Vatten, LJ 1436

Vaughan, L 1839

Vázquez, J 903

Veal, GJ 424, 725, 1675

Verheijen, RHM 1335

Verkooijen, HM 1743

Vermeulen, PB 1888

Vermorken, JB 1692

Vermund, SH 1480

Véronèse, L 474

Verrelle, P 474

Verschoyle, RD 248

Verspaget, HW 1896

Vestey, J 1772

Veyret, C 1633

Vezzosi, V 1253

Viale, G 1118

Victor, A 241

Vieillefond, A 336, 1314

Villari, P 1828

Vincent-Salomon, A 654

Visakorpi, T 352

Visioli, CB 1750

Vizoso, FJ 903

Vlastos, G 1743

Vogel, WF 808

Voigt, H 1540

Voltolini, L 1343

Vowler, SL 667

Vukmirović-Popović, S 1526

Vullierme, MP 49

Vuong, T 793

Wakefield, D 783

Waks-Yona, S 1667

Walch, AK 801

Walker, J 868

Walker, M 660

Walker, RA 639

Wall, L 868

Wallace, JA 591

Walter, S 1633

Waltering, K 352

Walton, M 29

Wanders, J 559

Wang, DK 623

Wang, H-W 1659

Wang, JH 262

Wang, R 821

Wang, Y 1579

Ward, RL 783

Ward, SE 1490

Warnberg, F 575

Watanabe, H 1191

Watanabe, M 1723

Waters, R 868

Watson, J 140

Watson, M 718

Watson, RWG 1587

Watson, SA 464, 1394, 1855

Weaver, FA 1083

Webb, EL 1904

Weber, WP 1072

Wee, A 776

Wehner, R 1293, 1928

Wei, WI 623

Weich, E 1699

Weiderpass, E 134

Weigle, B 1293, 1928

Weijenberg, MP 510

Weinman, J 1057

Welch, IM 95, 1377

Wells, A 1246

Welsh, FKS 1037

Wente, MN 1329

Wenz, F 912

Wespi, Y 1743

West, CML 95, 1377

Whang, EE 993

Wheelhouse, J 952

Whitchelo, N 95

White, D 575

White, J 29, 206

Whyman, G 424

Wicki, A 1

Wieland, W-F 1711

Wiklund, F 137

Wild, SH 1747

Willeke, F 912

Willems, R 1605

Willer, A 912

Williams, ARW 1613

Williams, GH 1384

Williams, MV 882

Wilson, C 891

Wilson, G 38

Wilson, JL 321

Winkler, C 296

Winqvist, R 352

Winslet, MC 1013

Winter, DL 1439

Wiuf, C 499

Wobser, M 1540

Wolk, A 1457

Wong, AS-C 776

Wong, VKH 222

Woo, JKS 617

Wood, R 752

Woods, LM 1135

Woodson, K 1587

Workman, P 29

Wotherspoon, A 1178

Wouters, BG 758

Wouters, D 769

Wray, NR 1613

Wright, D 875

Wu, T 262

Wyld, L 1025

Wyler, S 1072

Xiang, J 477

Xiao, ZJ 583

Xing, PX 918

Xu, H 1579

Yamaguchi, I 290

Yamamoto, H 986

Yamamoto, T 492

Yamazaki, K 400, 631

Yang, E 918

Yasutomi, M 1170

Yatabe, Y 1191

Yates, CC 1246

Yen, C-J 541

Yip, FK 126

Yokozaki, H 89

Yoshida, H 1549

Yoshida, S 1170

Yoshikawa, H 290

Yoshimura, S 1191

Young, A 732

Younis, T 162

Yu, J 1908

Yu, M 1659

Yu, MC 821

Yu, YL 623

Yuan, J-M 821

Yun, J-P 477

Zagonel, V 1639

Zaja, F 864

Zajac, P 1072

Zampino, MG 1118

Zandvliet, AS 559

Zappa, M 218, 1750

Zatonski, W 1450

Zeegers, MP 523, 1439

Zeillinger, R 485

Zerbib, M 336, 1314

Zhang, C-Q 477

Zhang, X 952

Zhao, P 172

Zhu, CQ 808

Zhu, T 583

Zilembo, N 439

Zlobec, I 793

Zong, YS 623

Zucchetta, P 1778

Zwaenepoel, O 692

Zweit, J 567

